# Full-thickness resection closure using reopenable-clip over-the-line method inside a submucosal pocket

**DOI:** 10.1016/j.vgie.2023.02.011

**Published:** 2023-04-12

**Authors:** Tatsuma Nomura, Shinya Sugimoto, Taishi Temma, Jun Oyamada, Keichi Ito, Akira Kamei

**Affiliations:** 1 Department of Gastroenterology, Mie Prefectural Shima Hospital, Shima, Mie, Japan; 2 Department of Gastroenterology, Ise Red Cross Hospital, Ise, Mie, Japan; 3 Department of Gastroenterology, Ise Red Cross Hospital, Ise, Mie, Japan

## Abstract

Video 1 Full-thickness defect resection closure using the reopenable-clip over-the-line method inside a submucosal pocket in the porcine stomach.

Full-thickness defect resection closure using the reopenable-clip over-the-line method inside a submucosal pocket in the porcine stomach.

## Description

**Figure 1:**
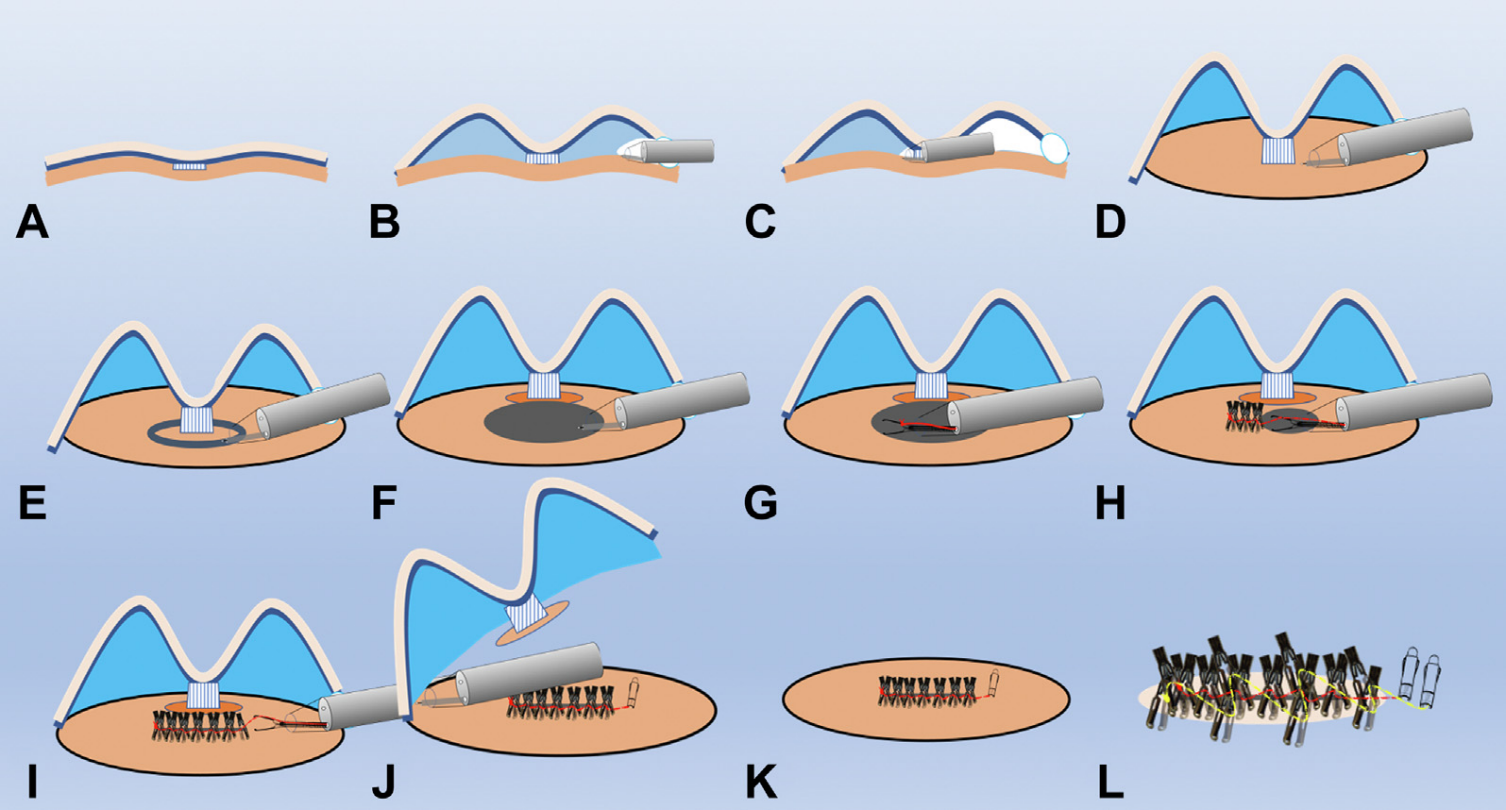
Schema of the reopenable-clip over-the-line method inside a submucosal pocket. **A-D,** A submucosal pocket is created, and the surrounding submucosa is dissected while excluding the center, creating a protrusion resembling a doughnut. **E and F,** Full-thickness resection is subsequently performed around the lesion, and the mucosal defect floor is perforated with a long axis of approximately 20 mm. **G-I,** A clip with a line is grasped and placed in the serous muscle layer on the anal side. Next, a reopenable-clip with a nylon line threaded through a tooth hole on one side is placed in the serous muscle layer (reopenable-clip over-the-line method). Finally, the reopenable-clip over-the-line method is repeated, allowing the full-thickness defect to be completely closed. **J,** The remaining mucosa is subsequently dissected. **K and L,** To prevent posterior bleeding, the reopenable-clip over-the-line method is repeated from the anal to the oral side to completely close the full-thickness defect.

Endoscopic submucosal dissection is an endoscopic technique that allows the complete resection of early tumors. A thin submucosal layer exists in gastric tumors with severe ulceration, and the muscle layer is thinning; therefore, cutting the muscle side for en bloc resection is necessary, and a safe resection method is desirable.^1^ However, surgery is the first choice when submucosa invasion is suspected, and submucosa-invasive carcinoma with a high risk of lymph node metastasis should not be treated with full-thickness resection.^2^ We previously reported the application of the reopenable-clip over-the-line method, which allows the complete closure of large, full-thickness defects in the muscle and serous layers.^3^, ^4^, ^5^, ^6^, ^7^, ^8^ The pocket-creation method provides ideal endoscopic maneuverability inside the submucosal pocket.^9^, ^10^, ^11^ Therefore, we developed a new procedure, termed the full-thickness resection-closure using reopenable-clip over-the-line method inside a submucosal pocket (ROLM-SP) (Fig. 1; Video 1, available online at www.videogie.org), which we demonstrate in a pig.

**Figure 2:**
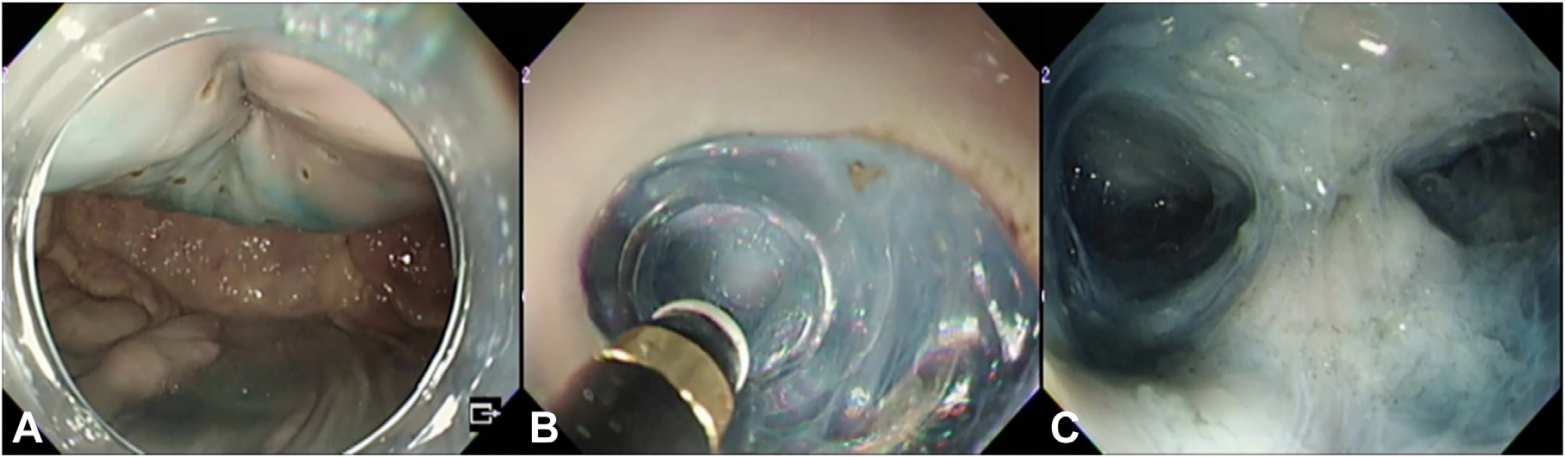
Creation of submucosal pockets outside of the full-layer lesions. **A,** A full-thickness lesion with a poorly elevated center after localization. **B,** The distance from the center and creation of the first mucosal incision. **C**, Dissection of the submucosa in the pocket, excluding the center.

**Figure 3:**
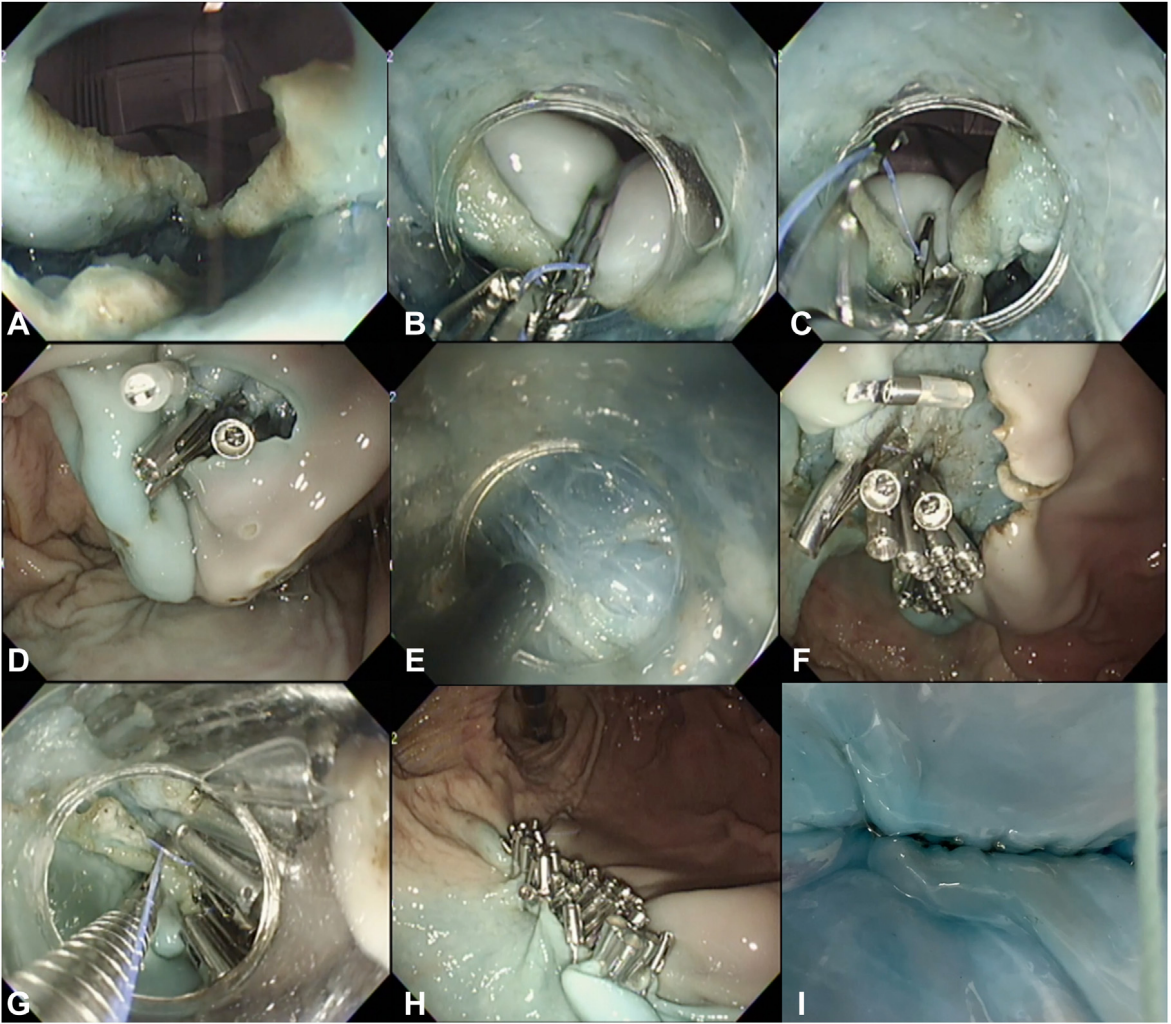
Actual procedure of the reopenable-clip over-the-line method inside a submucosal pocket. **A**, A 20-mm full-thickness defect after full-thickness resection in a submucosal pocket. **B,** Using the reopenable-clip over-the-line method, we placed a clip in the serous muscular layer, with the threaded teeth of the clip facing toward the defect. **C,** The serous muscular layer is completely closed by alternately placing the threaded clips. **D,** Full-thickness defect closure is completed, and the stomach is expanded by gas insufflation. **E**, Resection of the mucosa outside the remaining mucosa pocket. **F** and **G**, The remaining mucosal defects around the full-thickness closure. **H,** Second reopenable-clip over-the-line method, performed from the anal side. **I,** Full-thickness defect closure after the second reopenable-clip over-the-line method. **J,** No gas leakage was observed from the serosal side.

First, a full-thickness lesion was made with a thread in the middle of the pig’s stomach, and the surrounding area was marked (Fig. 2). Next, the first mucosal incision was made at a 20-mm distance from the center, and a calibrated, small-caliber tip, transparent hood with a 4-mm tapered tip was used to create a small mucosal incision. Subsequently, a submucosal pocket was created, and the surrounding submucosa was dissected while excluding the center, forming a doughnut-shaped protrusion. Full-thickness resection was performed around the lesion, and the mucosal defect floor was perforated with a long axis of approximately 20 mm (Fig. 3). All procedures were performed at low pressure to prevent gas leakage from the stomach wall because of excessive endoscopic insufflation. We used the reopenable-clip over-the-line method for full-thickness defect closure in the pocket. First, a clip with a line was grasped and placed in the serous muscle layer on the anal side. Next, a clip (SureClip, 8 or 16 mm; MC Medical, Tokyo, Japan) with a 0.22-mm nylon line threaded through a tooth hole on one side was placed in the serous muscle layer (reopenable-clip over-the-line method). The reopenable-clip over-the-line method was repeated, and the full-thickness defect was completely closed. To prevent the clips from becoming buried in the serosa, we temporarily grasped the serous muscle layer with the clips and pulled the line in hand to ensure that the clips were erect. The line was finally cut using a locking-clip technique, with the clip (EZ Clip, HX-610-090; OLYMPUS, Tokyo, Japan) fixing the line at the base of the clip and placed on the normal mucosa.^6^^,^^12^

Once the full-thickness defect was completely closed, insufflation was performed and the endoscopic view improved. The remaining mucosa was dissected, and the lesion was resected en bloc, with a long diameter of 40 mm and a 20-mm full-thickness resection. A leak test was performed, and almost no internal gas leaked from the closure. To prevent posterior bleeding, the reopenable-clip over-the-line method was repeated from the anal to the oral side, and the full-thickness defect was completely closed. No leakage from the stomach was observed, and the procedure time was 79 minutes. The total number of clips used in the procedure was 33.

When our ROLM-SP procedure is used, full-thickness resection can be performed with the pocket-creation method while maintaining low lumen pressure, followed by full-thickness closure of the serous muscle layer. The reopenable-clip over-the-line method is a feasible full-thickness defect closure technique because it does not require a single clip to grasp the bilateral defect edges.

## Disclosure

*The authors disclosed no financial relationships.*

## Supplementary data

Video 1 Full-thickness defect resection closure using the reopenable-clip over-the-line method inside a submucosal pocket in the porcine stomach.
